# Down-regulation of the cancer/testis antigen 45 (CT45) is associated with altered tumor cell morphology, adhesion and migration

**DOI:** 10.1186/1478-811X-11-41

**Published:** 2013-06-10

**Authors:** Anja Koop, Nadia Sellami, Sabine Adam-Klages, Marcus Lettau, Dieter Kabelitz, Ottmar Janssen, Hans-Jürgen Heidebrecht

**Affiliations:** 1 Institute for Immunology, University Hospital Schleswig-Holstein Campus Kiel, Arnold-Heller-Str. 3, Bldg 17, Kiel, 24105, Germany; 2 Institute for Pathology, University Hospital Schleswig-Holstein Campus Kiel, Arnold-Heller-Str. 3, Bldg 14, Kiel, 24105, Germany

## Abstract

**Background:**

Due to their restricted expression in male germ cells and certain tumors, cancer/testis (CT) antigens are regarded as promising targets for tumor therapy. CT45 is a recently identified nuclear CT antigen that was associated with a severe disease score in Hodgkin’s lymphoma and poor prognosis in multiple myeloma. As for many CT antigens, the biological function of CT45 in developing germ cells and in tumor cells is largely unknown.

**Methods:**

CT45 expression was down-regulated in CT45-positive Hodgkin’s lymphoma (L428), fibrosarcoma (HT1080) and myeloma (U266B1) cells using RNA interference. An efficient CT45 knock-down was confirmed by immunofluorescence staining and/or Western blotting. These cellular systems allowed us to analyze the impact of CT45 down-regulation on proliferation, cell cycle progression, morphology, adhesion, migration and invasive capacity of tumor cells.

**Results:**

Reduced levels of CT45 did not coincide with changes in cell cycle progression or proliferation. However, we observed alterations in cell adherence, morphology and migration/invasion after CT45 down-regulation. Significant changes in the distribution of cytoskeleton-associated proteins were detected by confocal imaging. Changes in cell adherence were recorded in real-time using the xCelligence system with control and siRNA-treated cells. Altered migratory and invasive capacity of CT45 siRNA-treated cells were visualized in 3D migration and invasion assays. Moreover, we found that CT45 down-regulation altered the level of the heterogeneous nuclear ribonucleoprotein syncrip (hnRNP-Q1) which is known to be involved in the control of focal adhesion formation and cell motility.

**Conclusions:**

Providing first evidence of a cell biological function of CT45, we suggest that this cancer/testis antigen is involved in the modulation of cell morphology, cell adherence and cell motility. Enhanced motility and/or invasiveness of CT45-positive cells could contribute to the more severe disease progression that is correlated to CT45-positivity in several malignancies.

## Background

Cancer/Testis (CT) antigens comprise a heterogeneous group of now more than 150 proteins with an eponymous expression pattern being restricted to male germ cells in normal human testis and to tumor cells of different origin [1-3]. CT antigens encoded on the X-chromosome form the subgroup of CT-X antigens [2]. Since several CT antigens induce specific cellular or humoral immune responses, they are regarded as promising targets for anti-tumor immunotherapy due to their absence from normal tissues [1,4,5]. In fact, fusion proteins or peptides derived from some of the first identified CT antigens such as MAGE-A3 and NY-ESO-1 are subject of present clinical phase II and III studies to evaluate their potential as cancer vaccines, e.g. for the treatment of myeloma [6-9]. Surprisingly, and also true for the CT antigens that were discovered already some 20 years ago, almost nothing is known about their function in developing germ cells or CT antigen-positive tumor cells [1,2].

The CT45 gene family was first identified in 2005 by signature sequencing and comprises 6 highly similar genes which are located on the X-chromosome (Xq26.3) [10]. CT45 is a nuclear protein with significant similarity to the CT-X antigen SAGE (CT14) and the D-E-A-D box containing protein DDX26 [10]. In normal human tissues, CT45 expression is restricted to spermatogonia and spermatocytes. Many human tumors do not express CT45 at all. In some tumors, e.g. colon carcinoma, CT45 is expressed in a low number of cases (10%). Only in germ cell tumors (e.g. seminoma), in Hodgkin’s lymphoma, ovarian cancer and multiple myeloma, CT45 is expressed in a larger number of cases [11-15]. Similar to other CT antigens, CT45 gene expression is epigenetically controlled by methylation [6,16,17]. Thus, methylated CpG islands in the CT45 promotor suppress CT45 expression, whereas demethylation by 5′-aza-2′-deoxycytidine treatment induces the expression of CT45 even in *a priori* CT45-negative HeLa cells [12] (and own unpublished results).

At the protein level, CT45 migrates as a double band of 22/25 kDa after immunopurification and/or Western blotting [12]. Initial immunocytochemical analyses using the anti-CT45 mab Ki-A10 revealed that CT45 is exclusively found in the nuclei, with a strong enrichment in so-called nuclear speckles [18]. Evaluation of a large panel of Hodgkin‘s lymphoma with this monoclonal antibody facilitated the discrimination of Hodgkin's lymphoma from lymphadenopathies. Moreover, a high expression of CT45 correlated with more aggressive histological subtypes, B symptoms (e.g. fever, night sweats, and weight loss) and advanced stages, indicating that CT45 might serve as a marker for a worse course of Hodgkin’s lymphoma [19,20]. Similarly, in a recent independent study, poorer prognosis and outcome were also demonstrated for multiple myeloma patients with CT45-positive tumors as compared to CT45-negative specimen [13].

Thus, CT45 has already proven its relevance as a potential prognostic marker for several types of tumors [13,19,20]. Its association with disease progression, severity and poor prognosis suggests that CT45 might somehow support tumor cell malignancy or aggressiveness, as has been proposed for other CT antigens. For instance, altered cell proliferation and/or motility were observed upon down-regulation of members of the MAGE-family in mast cells or multiple myeloma cells and SSX in mesenchymal stem cells or by ectopic overexpression of CAGE in fibroblasts [21-24].

In order to assess the cell biological function of CT45, we investigated the effects of CT45 down-regulation by RNA interference in established CT45-positive Hodgkin’s lymphoma, myeloma and fibrosarcoma cell lines. We report that reduced levels of CT45 do not alter cell cycle progression or proliferation, but modulate cell morphology, adherence and migration as shown for Hodgkin’s lymphoma and/or fibrosarcoma cell lines. We suggest that altered cell morphology, enhanced motility and invasiveness of CT45-positive cells might contribute to the higher degree of malignancy that has been associated with CT-positive Hodgkin’s lymphoma and multiple myeloma.

## Results

### Subcellular localization of CT45 and down-regulation of CT45 by RNA interference

**Figure 1:**
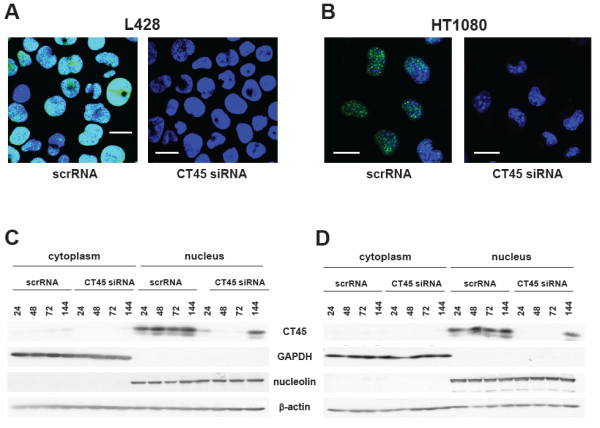
**Subcellular localization of CT45 and down-regulation of CT45 by RNA interference.** Immunofluorescence staining of cytospin preparations of L428 cells (**A**) and of adherently growing HT1080 cells (**B**) treated with scrambled (scrRNA) or with CT45-specific (CT45 siRNA) with mab Ki-A10. DAPI was used for DNA staining. Down-regulation of CT45 using CT45 siRNA was analyzed after 72 h (**A**, **B**) or over a period of up to 144 h in L428 (**C**) and HT1080 cells (**D**). Cytoplasmic and nuclear fractions of the cells were prepared and CT45 expression was analyzed with mab Ki-A10 in L428 and mab Ki-CT45-2 in HT1080 cells. β-actin staining was performed to demonstrate equal loading, GAPDH was used as a cytoplasmic and nucleolin as a nuclear marker. All experiments were performed at least three times. White bars indicate 20 μm.

CT45-positive Hodgkin’s lymphoma (L428), fibrosarcoma (HT1080) and myeloma (U266B1) cells were examined by immunofluorescence staining with the CT45-specific mab Ki-A10. We observed a dot-like nuclear expression of CT45 in the suspension cell lines L428 (Figure 1A) and U266B1 (Additional file 1: Figure S1A) and also in adherently growing HT1080 cells (Figure 1B). Immunofluorescence staining of L428 or HT1080 cells transfected with either scrRNA or CT45-specific siRNA was performed using mab Ki-A10 72 h after transfection. SiRNA-treated L428 or HT1080 cells showed an almost complete down-regulation of CT45 protein (Figure 1A, B). Down-regulation of CT45 by specific RNA interference resulted in a clear reduction of CT45 protein levels in the tested cell lines between 24 h to 96 h after transfection (Figure 1C, D, supplemental Figure 1B). Cell lysates of scrRNA- or siRNA-treated cells were prepared 24 to 144 h after transfection and analyzed by Western blotting. The predominant nuclear distribution was verified by probing cytoplasmic and nuclear extracts of L428 and HT1080 cells. CT45 was detected in the nuclear fraction but not in the cytoplasm (Figure 1C, D) where GAPDH served as a marker for the cytosolic and nucleolin for the nuclear fraction. Of note, for detecting CT45 from L428 cells, we used mab Ki-A10 and from HT1080 cells mab Ki-CT45-2. Although CT45 was detected in HT1080 cells by immunofluorescence staining using mab Ki-A10, it was barely visible in lysates of HT1080 cells using this antibody, presumably due to a significantly lower overall protein abundance as was indicated by the immunofluorescence staining. Interestingly, however, the newly established anti-CT45 antibody Ki-CT45-2 that is directed against an N-terminal epitope of CT45 (data not shown) also stained the protein in HT1080 lysates.

### Down-regulation of CT45 does not interfere with proliferation

**Figure 2:**
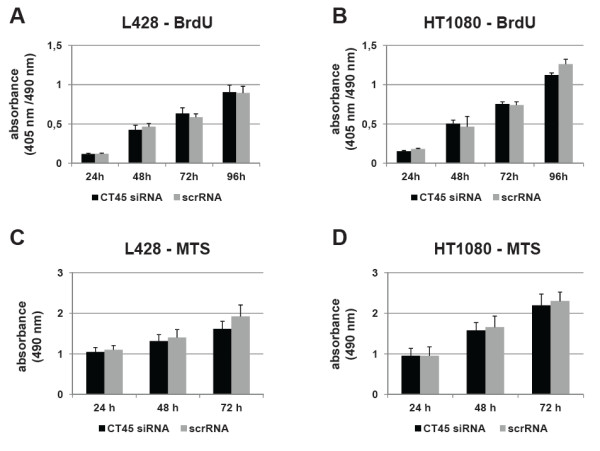
**Impact of CT45 down-regulation on cell proliferation.** BrdU (**A**, **B**) and MTS (**C**, **D**) assays were performed in quadruplicates to determine the effect of CT45 down-regulation on cell metabolism and proliferation of L428 (**A**, **C**) and HT1080 (**B**, **D**) cells. Data are shown for representative experiments of two to six independent assays. In all cases, inhibition of CT45 expression had no significant effect on cell proliferation and cell growth in the tested cell lines.

**Figure 3:**
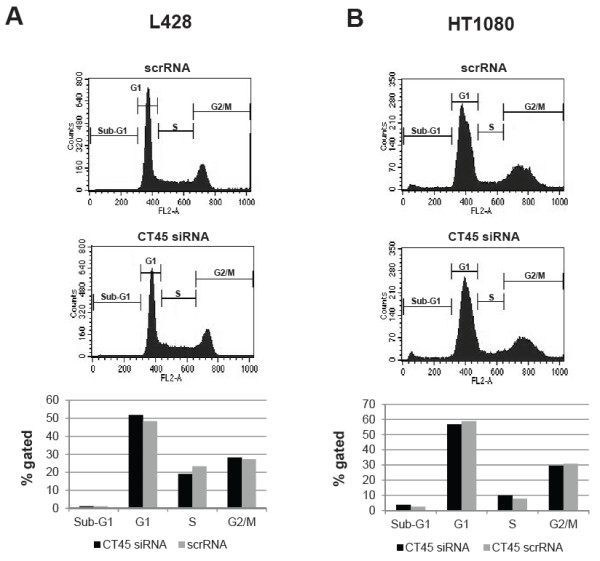
**Impact of down-regulation of CT45 on cell cycle progression.** 72 h after transfection with scrRNA or CT45 siRNA, cells were harvested and labeled with propidium iodide. The analysis of the different cell cycle phases of L428 (**A**) and HT1080 (**B**) cells was performed by flow cytometry. Representative histograms for scrRNA- or CT45 siRNA-treated cells are shown in the upper and middle panels. A quantification using the indicated regions is depicted in the lower panels. Down-regulation of CT45 had no apparent impact on cell cycle progression. All experiments were performed at least three times.

To assess whether the knock-down of CT45 would influence the autonomous proliferation of L428, HT1080 or U266B1 cells, we performed BrdU incorporation assays to analyze DNA synthesis (Figure 2A, B, supplemental Figure 1C) and MTS assays to measure metabolic activity (Figure 2C, D). In contrast to what has been reported for several other CT antigens, the specific down-regulation of CT45 did not have any apparent effect on the proliferation of the tested tumor cells. No significant differences between control cells and siRNA-treated cells were observed at any time point. Furthermore, siRNA- and scrRNA-treated L428 (Figure 3A), HT1080 (Figure 3B) and U266B1 (supplemental Figure 1D) cells were collected at different time-points and subjected to cell cycle analysis by flow cytometry using the DNA dye propidium iodide. One representative experiment performed 72 h after transfection is shown for each cell line. As calculated from the individual FACS profiles shown in the upper panels, there was no detectable effect on the distribution of cells in the different phases of the cell cycle (Figure 3A, B, supplemental Figure 1D, lower panels).

### CT45 knock-down alters morphology

**Figure 4:**
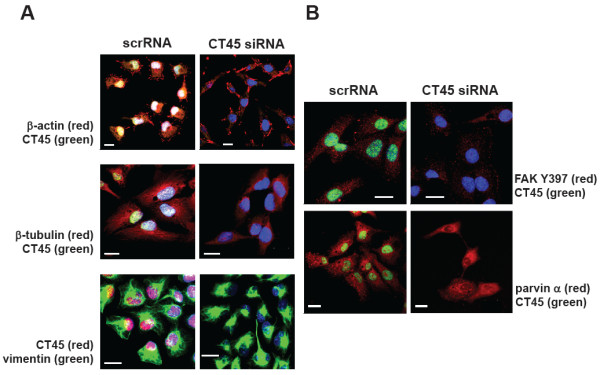
**Down-regulation of CT45 alters cell morphology.** (**A**) 72 h after transfection, double immunofluorescence staining of adherently growing HT1080 cells with mab Ki-A10 and antibodies specific for actin, ß-tubulin and vimentin was performed. ScrRNA-treated cells demonstrate normal cell morphology, whereas CT45 siRNA-treated cells show a significantly altered cell morphology (right panels in **A**). (**B**) Double immunofluorescence staining of focal adhesion proteins FAK Y397 and parvin alpha was performed in HT1080 cells 72 h after siRNA transfection. Focal adhesion sites in scrRNA-treated cells are indicated by arrows. Down-regulation of CT45 resulted in a markedly reduced number of FAK Y397- and parvin α-positive focal adhesion sites. White bars indicate 20 μm.

On the basis of our observation that siRNA-treated HT1080 cells were less adherent on FCS-coated glass slides compared to control cells, we analyzed the intracellular distribution of characteristic cytoskeletal elements. Since morphological changes were best seen in HT1080 cells, we performed the next series of experiments with the adherently growing fibrosarcoma cell line. ScrRNA- and siRNA-treated HT1080 cells were stained 72 h after transfection with antibodies specific for actin, microtubules and intermediate filaments (Figure 4A), or specific for marker proteins for focal adhesion proteins (Figure 4B). Double immunofluorescence staining of scrRNA-treated control cells with Ki-A10 and antibodies specific for actin, ß-tubulin and vimentin reflected the normal morphology of adherently growing HT1080 cells. In contrast, CT45 down-regulated cells revealed an altered morphology as indicated by changes in the cell shape (e.g. upper panels, ß-actin staining) and the organization of the microtubule network (middle panels, ß-tubulin staining) which appeared more diffuse and unorganized in siRNA-treated cells. Similar to actin, also the staining for vimentin, a marker protein of intermediate filaments, pointed to major alterations in cell shape and size. Moreover, the comparison of scrRNA- and siRNA-treated cells using antibodies specific for phosphorylated focal adhesion kinase (FAK Y397) and parvin alpha - both proteins play an important role in the regulation of cell adherence - showed a markedly reduced number of active focal adhesion sites in siRNA-treated cells (Figure 4B).

### CT45 repression alters adherence, migration and invasion

**Figure 5:**
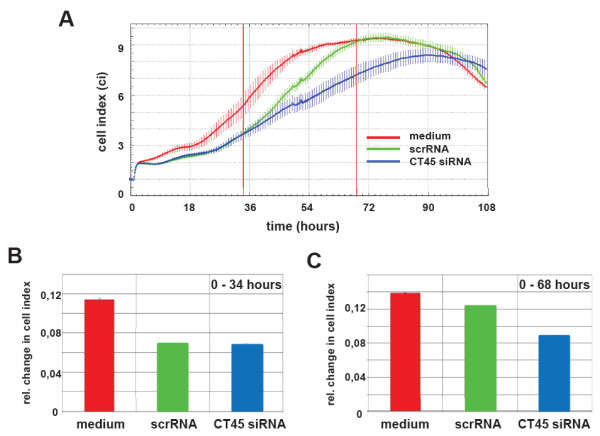
**Influence of CT45 down-regulation on the time-dependent adherence profiles of HT1080 cells.** (**A**) To analyze the effect of down-regulation of CT45 on cell adhesion, we performed real-time measurements of the cell impedance over a period of 108 h with HT1080 cells as a control (red line), scrRNA-transfected cells (green line) and CT45 siRNA-transfected cells (blue line). Starting after 34 h, the cellular impedance of CT45 siRNA-treated cells decreased significantly reaching a maximal difference (18%) at 68–72 h after transfection. The changes in the cell indices were calculated for the time intervals 0–34 h (**B**) and 0–68 h (**C**), respectively using the RTCA-integrated software provided by the manufacturer. Data of one representative experiment (with four individual biological replicates) of four are shown.

Having shown that the cytoskeletal composition and the formation of cell contacts were altered in the presence of CT45, we next analyzed its potential impact on cell adherence and motility. First, a real-time analysis of cell adherence was performed using the xCelligence system from Roche. With this assay system, we recorded alterations of the cellular impedance of *in vitro* cultured scrRNA- and siRNA-treated HT1080 cells over time (Figure 5A). As expected, the transfected cells displayed a slightly decreased cell index as compared to untransfected cells. During the first 34 h after transfection, no difference in the cellular impedance was seen between scrRNA- and CT45 siRNA-treated cells (Figure 5B). After 34 h, however, the cellular impedance of siRNA-treated cells decreased, reaching a maximal difference at 68–72 h after transfection (Figure 5C). To ensure that the observed effects on cellular impedance were not due to changes in proliferation, both cell populations were labeled with BrdU 34 h after transfection. As expected, we did not detect alterations in the proliferative capacity in the presence or absence of CT45 (data not shown). Since cellular impedance in this system is influenced by the number of cells and their individual adherence, we concluded that CT45 down-regulation reduced the adherence of siRNA-treated HT1080 cells.

**Figure 6:**
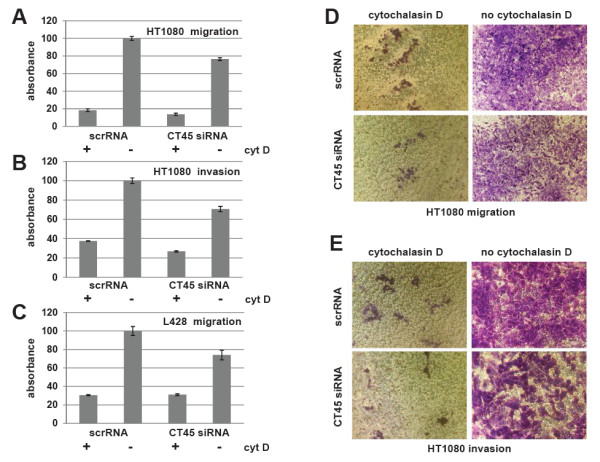
**CT45 affects cell invasion and migration.** (**A-C**) Cell migration and cell invasion capacities of scrRNA- or CT45 siRNA-treated HT1080 or L428 cells were analyzed in colorimetric or fluorometric transwell systems as described in the experimental procedures (**A**, **D**: HT1080 migration, **B**, **E**: HT1080 invasion, **C**: L428 migration). As a control, cell motility was inhibited with 2 μM Cytochalasin D. The data analysis revealed that cell migration of siRNA-treated HT1080 cells was down-regulated by 23%. Staining of cells collected from the lower chamber revealed that less siRNA-transfected and only very few cytochalasin D-treated cells had passed the membrane (**D**). The cell invasion assay of HT1080 (**B**, **E**) and the migration assay for L428 cells (**C**) revealed similar results. All experiments were performed at least three times.

We next tested the migratory and invasive capacity of scrRNA- and CT45 siRNA-treated HT1080 cells using transwell migration and invasion assay systems. All analyses were performed 48 h after down-regulation of CT45 using cytochalasin D-treated HT1080 cells as an internal control. The cells were placed in the upper chamber of the transwell system and incubated for 4 h before staining and quantification. The data analysis revealed that cell migration of siRNA-treated HT1080 cells was down-regulated by 23% (Figure 6A, D). In addition, staining of cells collected from the lower chamber revealed that less siRNA-transfected and only very few cytochalasin D-treated cells had passed the membrane. A subsequent analysis of HT1080 cells in a modified cell invasion assay confirmed the previous results (Figure 6B, E). Moreover, also the cell migration of the non-adherent CT45-positive Hodgkin’s lymphoma cells L428 was significantly reduced upon siRNA-treatment in a separate migration assay (Figure 6C).

### Potential impact of CT45-regulated syncrip on cell morphology

**Figure 7:**
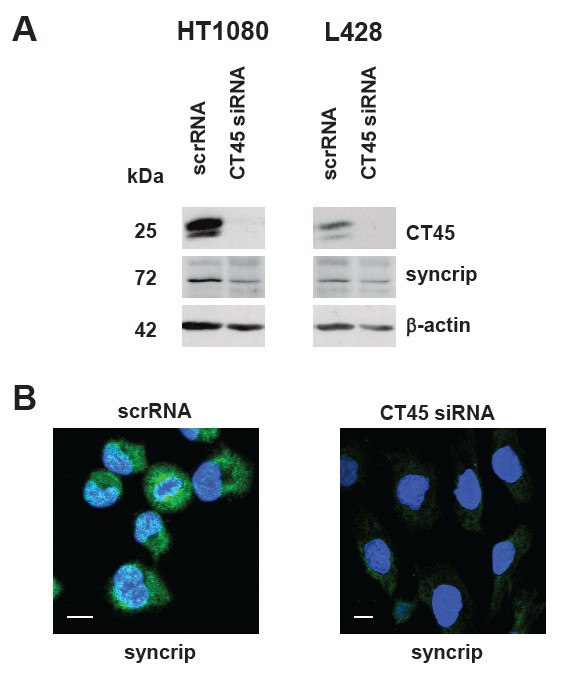
**CT45 influences the expression of syncrip.** (**A**) L428 and HT1080 cells were transfected with scrRNA or CT45 siRNA, respectively. 72 h after transfection, Western blot analyses of cell lysates were performed using antibodies specific for CT45 (Ki-A10 for L428 cells, and Ki-CT45-2 for HT1080 cells), syncrip and ß-actin (as loading control). 72 h after down-regulation of CT45, syncrip is also down-regulated in both cell lines. (**B**) In parallel, immunofluorescence staining for syncrip of scrRNA-transfected and CT45 siRNA-transfected HT1080 cells was performed. DAPI was used for visualization of the nuclei. White bars indicate 20 μm.

2D-DIGE experiments comparing scrRNA- and CT45 siRNA-treated cells revealed that syncrip (hnRNP-Q), a mRNA-binding heterogeneous nuclear ribonucleoprotein involved in the control of focal adhesion formation and cell motility [25], is partially down-regulated 72 h after CT45 siRNA transfection of HT1080 and L428 cells (unpublished data, PhD thesis of Anja Koop, CAU, Kiel). Western blot analyses of lysates of HT1080 and L428 cells obtained 72 h after CT45 siRNA transfection confirmed these results (Figure 7A). Along with the down-regulation of CT45 (demonstrated by staining with mabs Ki-A10 and Ki-CT45-2, respectively), also syncrip was reduced in siRNA-treated cells. Immunofluorescence staining of HT1080 cells with an hnRNP-Q-specific mab showed a weak dot-like nuclear expression and a strong cytoplasmic staining in scrRNA-treated cells (Figure 7B, left panel). In contrast, in CT45 siRNA-treated cells using the same settings, the cytoplasmic staining was much less prominent suggesting that CT45 somehow regulates the subcellular distribution of syncrip (Figure 7B, right panel).

**Figure 8:**
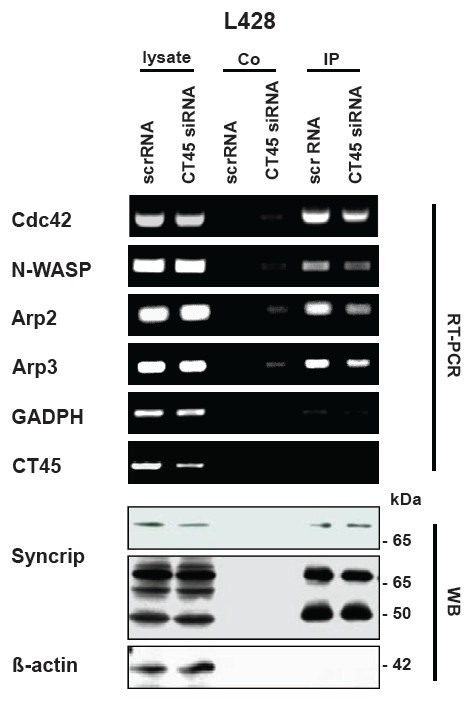
**Syncrip selectively co-precipitates mRNAs of Cdc42, N-WASP, Arp2, Arp3 but not GAPDH and CT45.** L428 cells were transfected with scrRNA or CT45 siRNA, respectively. 72 h after transfection, cell lysates were prepared and syncrip immunoprecipitations performed as detailed in Material and Methods. Protein A beads used for preclearing were used as internal control (co) for the RT-PCR or Western blot. In addition, respective RT-PCRs were also performed using unseparated cell lysates as starting material. The whole cell lysates were also used to document the mild reduction of syncrip protein by Western blotting (WB, upper panel for syncrip, short exposure; lower panel, long exposure). The RT-PCR revealed that mRNAs for Cdc42, N-WASP, Arp2 and Arp3 (but not CT45 or GAPDH) could be amplified from syncrip-immunoprecpitations. In addition, the amount of co-precipitated mRNA was reduced in CT45 siRNA-treated cells.

Regarding the regulatory function of syncrip on morphogenesis, recent reports suggest that syncrip might interact with several mRNAs encoding for key proteins of cytoskeletal reorganization including RhoA, Cdc42, N-WASP and constituents of the Arp complex [26,27]. In neurons, the absence of syncrip results in an inappropriate localization of these proteins and in abnormal neurite arborization and morphological alterations. We therefore analyzed whether syncrip would also bind to respective mRNAs of CT45-positive tumor cells and whether the knock-down of CT45 would alter the amount of precipitated mRNA. To this end, we performed mRNA-co-immunoprecipitations from L428 cells (Figure 8). We detected equal levels of individual mRNAs in the lysates of scrRNA- and CT45 siRNA-treated cells. However, when we precipitated syncrip from these lysates, in correlation to the reduced level of syncrip, we also observed a reduction of co-precipitated mRNA for Cdc42, N-WASP, Arp2 and Arp3. With these initial hints, we suggest that also in the investigated tumor cells, the reduction of CT45 alters the syncrip-driven regulation of cytoskeletal elements.

## Discussion

To assess the relevance of CT45 in tumor cells, we analyzed L428 (Hodgkin’s lymphoma), U266B1 (myeloma/plasmacytoma) and HT1080 (fibrosarcoma) cells. Although these cell lines differ in their origin and morphology, e.g. HT1080 cells being adherent and L428 and U266B1 cells non-adherent, they were used because they homogenously express CT45. In all three cell types, a strong nuclear expression of CT45 was detected with a punctate enrichment in subnuclear structures reminiscent of nuclear speckles. It is believed that nuclear speckles are dynamic structures that form storage sites of pre-mRNA splicing factors [28]. Although off-target effects are more and more discussed, the inhibition or down-regulation of proteins by RNA interference has proven to be an excellent tool to obtain unbiased information about the function of otherwise poorly characterized proteins in many instances, also including studies on CT antigens [21,22,29]. Using CT45-specific siRNA, a substantial down-regulation of CT45 was visible 24 h after transfection of HT1080, U266B1 and L428 cells with an almost complete knock-down after 72 to 96 h. Down-regulation was transient, since 144 h after transfection, CT45 expression was again detectable.

Altered proliferation, cell morphology, adherence, migration and invasion are typical characteristics of cancerous cells [24,30]. It was shown before that higher levels of the cancer/testis antigens CT7 and MAGE-A3/6 correlated with increased plasma cell proliferation [23], and that down-regulation of members of MAGE-A, -B, and -C families by siRNA reduced cell proliferation of human HMC1 and murine P815 mast cells. In addition, these two cell lines displayed a prolonged S-phase 24 h after siRNA transfection [21]. Moreover, Por and coworkers described that the down-regulation of the cancer/testis antigen CAGE by RNA interference retarded cell proliferation in HeLa and Malme-3 M melanoma cells [22]. Therefore, we initially analyzed the effects of CT45 down-regulation on the proliferation of L428, HT1080 and U266B1 cells. However, although we used different approaches to assess cell proliferation, we did not observe any alterations in cell growth upon CT45 knock-down. In addition, we also did not detect any impact on cell-cycle progression throughout the observation time of up to seven days.

In the course of our studies, however, we noted apparent differences in cell adherence and morphology upon siRNA-treatment of adherent HT1080 cells. Changes in the intracellular distribution of key cytoskeletal elements were demonstrated by immunofluorescence staining for actin, ß-tubulin and vimentin. Here, scrRNA-treated cells showed a characteristic morphology with needle-like filopodia. In contrast, in siRNA-treated cells, filopodia formation seemed to be impaired. The analysis of marker proteins for cell adherence such as the focal adhesion kinase (FAK) revealed several focal adhesion sites on control cells but much less upon CT45 down-regulation. In this context, Kim and colleagues recently reported that overexpression of the cancer/testis antigen CAGE enhanced the cell motility of cancer cells [31]. Phosphorylated FAK was significantly overexpressed in CAGE-expressing cells. Thus, our immunofluorescence analyses pointed to a first potential function of CT45 in the regulation of cell adhesion and migration.

It is evident that cell migration plays a pivotal role in a wide variety of biological processes including embryogenesis and cancerogenesis or metastasis formation [32]. Recently, Cronwright and colleagues demonstrated that down-regulation of the CT antigen SSX affected cell migration of a melanoma cell line, indicating that also SSX might support cell migration of certain tumor cells [24]. Along this line, the cell migration and invasion assays that we performed with HT1080 and L428 cells clearly indicated that CT45 is involved in the regulation of both cell migration and invasiveness. Since enhanced migration and invasion may contribute to an increased metastatic potential of CT45-expressing cells, this might in part explain the more severe disease progression and poorer prognosis in patients with CT45-positive tumors. It is noteworthy in this context that also tumor-associated non-CT antigens such as for example the neuronal transmembrane cell adhesion molecule L1CAM (CD171) have a strong impact on cell migration and invasion when “re-expressed” in treatment-resistant forms of ovarian and pancreatic cancer (while being absent from normal tissues) [33]. Even more interesting, the localization of the L1 gene on the X-chromosome (Xq28) and also its (de-)regulation by hypomethylation, very much resembles what has been observed for several CTX, including CT45. Since in a very recent paper, Bert and colleagues described long-range epigenetic remodelling as a mechanism for regional activation of a cancer genome [34], this might in part explain the new appearance of ‘mislocated’ protein products from otherwise silenced genes.

The obtained information, however, provokes the question of how nuclear CT45 could alter cell adherence, morphology and invasiveness. Here, we demonstrate that the CT45 knock-down coincides with a down-regulation and altered distribution of the ribonuclear protein syncrip (hnRNP-Q). Until now, three functionally active isoforms of syncrip are known (hnRNP-Q, -Q2 and -Q3) [25]. Apparently, two of these isoforms are predominantly located in the nucleus and are required for efficient pre-mRNA splicing whereas the third isoform is mostly found in the cytoplasm. Xing and colleagues recently reported that down-regulation of hnRNP-Q induced an up-regulation of the small GTPase RhoA causing morphological changes in murine C2C12 myoblastoma cells [26]. Furthermore, a reduction of syncrip induced evident morphological changes in rat cortical neurons and mouse neuroblastoma cells by regulating actin dynamics via the Cdc42/N-WASP/Arp2/3-pathway [27]. Therefore, it was concluded that syncrip is involved in mRNA processing to regulate transcription of cytoskeleton-regulatory proteins. Thus, being localized in a common nuclear compartment, syncrip could form the functional link between CT45 and the observed alterations in cell morphology and migration observed in our study. To support this hypothesis, the mRNA co-precipitation with syncrip revealed reduced levels of Cdc42, N-WASP, Arp2 and Arp3 mRNAs from CT45 siRNA-treated L428 cells. In line with the report by Xing and colleagues for neurons, in preliminary experiments, we observed a mild increase in RhoA when syncrip was reduced due to the CT45 knock-down. Moreover, we noted a slightly increased phosphorylation of cofilin arguing that changes might also occur at the level of postranslational modification of individual cytoskeletal proteins (data not shown). However, more extensive studies are needed to address how the changes in mRNA levels are eventually translated into the observed morphological changes. It will be important to analyze cytoskeletal or motility-promoting protein complexes in the presence or absence of CT45 and/or syncrip and to address changes in the subcellular localization or post-translational modifications.

## Conclusion

We provide first experimental evidence for a cell biological function of the cancer/testis antigen 45. We demonstrate that CT45 is involved in the regulation of cell morphology, adherence and migration. Enhanced motility and/or invasiveness of CT45-positive cells could be advantageous for spreading or metastasis formation and thereby contribute to the higher degree of malignancy or aggressiveness that has been associated with CT45-positive tumor cells.

## Material and methods

### Cells

The cell lines L428 (Hodgkin’s lymphoma, catalogue code ACC-197, German Collection of Microorganisms and Cell Cultures (DSMZ) Braunschweig, Germany), U266B1 ([U266] myeloma/plasmacytoma, ATCC® TIB-196™) and HT1080 (fibrosarcoma, catalogue code ACC-315, DMSZ) were grown in RPMI 1640 or DMEM, respectively. Media were supplemented with 10% fetal calf serum, 10 mM glutamine, 25 mM HEPES and 50 μg/ml streptomycin and penicillin.

### Immunofluorescence microscopy

For immunofluorescence staining, cytospin preparations of L428, U266B1 or HT1080 cells growing on fetal calf serum (FCS)-coated glass slides were fixed for 10 minutes in ice-cold methanol. The CT45-specific mab Ki-A10 was used for immunofluorescence staining [11,12]. Antibodies specific for syncrip (hnRNP-Q), actin and parvin alpha were purchased from Abcam (Cambridge, UK), for vimentin and focal adhesion kinase phosphorylated on tyrosine 397 (FAK Y397) from Cell Signaling Technology (Danvers, MA, USA) and for ß-tubulin from Sigma-Aldrich (Munich, Germany). Unlabeled mab were stained with donkey anti-mouse Alexa 488 or donkey anti-rabbit Alexa 594 (Invitrogen, Karlsruhe, Germany). DNA staining was performed with DAPI (Invitrogen). Confocal laser scanning microscopy was performed using an LSM 510 Meta microscope (Carl Zeiss, Jena, Germany).

### Silencing of CT45 in HT1080, U266B1 and L428 cells by RNA interference

Out of six different siRNAs that were initially tested, only one siRNA sequence (sense 5′-3′ GGAGAGAAAAGGAUCAGAUUU) was able to target the CT45 transcript effectively. For control purposes, a scrambled sequence was generated and verified (scrRNA 5-3′CUCGACAUAACACUGGUGCUU). The RNAs were purchased from Ambion/Applied Biosystems (Austin, TX, USA) and used at a concentration of 100 nM. Transfection was performed by electroporation using Nucleofector™ II equipment and nucleofector Kits for L428 and U266B1 (Kit L) and HT1080 cells (Kit T; Lonza, Cologne, Germany).

### Western blot analysis

After intense washing with ice-cold PBS, cells were lysed with 2% Triton-X 100, 1 mM EDTA and a protease inhibitor cocktail (Roche, Mannheim, Germany) in PBS for 2 minutes. Enriched cell nuclei were pelleted and then lysed with the same lysis buffer for 20 minutes. The enriched nuclear lysate was centrifuged at 15.000 × g. The resulting supernatant was boiled in loading buffer under reducing conditions and separated by SDS-PAGE. Immunostaining of blotted proteins was performed as described [35]. For some experiments, the ProteoJET™ Cytoplasmic and Nuclear Extraction Kit (Fermentas, St. Leon-Rot, Germany) was used following the manufacturer’s protocol.

### Cell proliferation and metabolic activity

Metabolic activity / proliferation of HT1080, U266B1 and L428 cells was measured by two colorimetric immunoassays according to the manufacturer’s protocols (BrdU-Assay Kit; Roche, Mannheim, Germany; MTS assay, Promega, Mannheim, Germany). HT1080 cells were seeded at a density of 5×10^3^ cells/well, L428 and U266B1 at a density of 2×10^4^ cells/well into 96-well flat bottom tissue culture plates. The total incubation period after transfection was 72 h (MTS) or 96 h (BrdU) and the metabolic activity or cell proliferation was measured every 24 h. For quantification, a microplate spectrophotometer was used with a detection wavelength of 490 nm for the MTS assay and detection at 490 nm with reference to 405 nm for the BrdU assay.

### Cell cycle analysis

ScrRNA- and siRNA-treated L428, U266B1 and HT1080 cells were washed with PBS and fixed in 50% ethanol at 4°C. After 30 min of fixation, the cells were washed twice in PBS, suspended in 0.1 ml PBS containing 40 μg/ml RNase A and incubated for 30 min at room temperature before 0.5 ml staining solution (50 μg/ml propidium iodide in PBS/5 mM EDTA) was added. Cell cycle analysis was performed by flow cytometry using a FACSCalibur Analyzer (Becton Dickinson)*.*

### Real-time monitoring of HT1080 cells using the xCELLigence system

The xCELLigence system (Roche Applied Science, Mannheim, Germany) monitors cellular events in real-time by recording the electrical impedance that is correlated with cell number, morphology and viability in a given culture well. For analyzing HT1080 cells after CT45 knock-down, we used 96 well microtiter E-plates. Fifty μl of culture medium were applied to determine the background impedance (30 min). Then, untransfected, and individually scrRNA- or CT45 siRNA-transfected HT1080 cells were adjusted to 200.000 cells/ml and 50 μl of each cell suspension was pipetted to three or four wells of the E-plate before placing it into the RTCA SP Station. Cell impedance was monitored every five minutes for the first hour and every ten minutes for the next 108 hours. The electrical impedance was calculated by the RTCA-integrated software of the xCELLigence system as a dimensionless parameter termed CI. Median values and standard deviations were calculated from four (in some experiments three) individual wells reflecting four (three) individual controls, scrRNA- or CT45 siRNA-transfections. The changes in cell indices for the time intervals 0–34 h and 0–68 h were also calculated by the RTCA-integrated software.

### Transwell invasion and migration assays

To determine the migratory and invasive potential of HT1080 cells after CT45 knock-down, we used the CytoSelect™ 24-well cell migration and invasion assay, colorimetric format (8 μm-pore size, CBA-100-C) from Cell Biolabs Inc. (San Diego, USA). In addition, cell migration of L428 cells was analyzed with the CytoSelect™ 24-well cell migration assay, fluorometric format (5 μm-pore size, Cat. CBA-102). The assays were performed as described in the manufacturer´s protocol. In brief, cells were washed once in serum free medium and seeded into the upper chamber onto a rehydrated basal layer membrane covering a matrigel preparation with a diameter of 8 μm (HT1080 cells) or 5 μm (L428 cells), respectively. For negative controls, 2 μM cytochalasin D was added. To investigate the invasive potential of scrRNA- and CT45 siRNA-transfected HT1080 cells, the cells were allowed to invade for 24 h. The migration capability of HT1080 cells was analyzed after 4 h and of L428 cells after 6 h of incubation. HT1080 cells on the bottom of the membrane were stained according to the manufacturer’s protocol, visualized with a light microscope and quantified as described in the assay protocol. L428 cells were removed from the lower surface of the membrane and all cells in the lower chamber were labeled and measured following the manufacturer’s instructions. All assays were repeated at least three times.

### mRNA-immunoprecipitation

For mRNA-immunprecipitation, 10^7^ L428 cells were lysed in 1 ml RSB100-buffer (10 mM Tris pH 7.4, 100 mM NaCl, 2.5 mM MgCl_2_) with 0.5 mg/ml digitonin for 20 minutes at 4°C. Lysates were centrifuged for 5 min at 1500 × g and the supernatant was further centrifuged for 15 min at 4000 × g. The resulting supernatant was then pre-cleared with 100 μl RSB100-buffer/protein-A-sepharose beads (50% (v/v), these beads were washed and used as controls). For mRNA-IP, the samples were incubated with 5 μg anti-syncrip antibody (Abcam) and protein-A-sepharose beads in RSB100-buffer for 2 hours. Pelleted beads were washed thrice with NET-2 buffer (50 mM Tris pH 7.4, 150 mM NaCl) with 0,5% Triton X-100. Finally, an equal volume of RSB100-buffer was added to the protein-A-sepharose and the samples were divided into two aliquots for mRNA isolation, cDNA synthesis and PCR or Western blotting, respectively. As a positive control, mRNA was also isolated from whole cell lysates. For mRNA isolation, the RNeasy Kit from Qiagen (Hilden, Germany) was used and for cDNA synthesis, the First Strand cDNA Synthesis Kit from Fermentas according to the manufacture's instructions. Cdc42, N-WASP, Arp2, Arp3, GAPDH and CT45 sequences were amplified from 2 μg cDNA using Dream Taq™ Polymerase (5 U/μl, Fermentas) and the primer pairs listed in Table 1. Fragments were analyzed on agarose-gel (1%) with ethidium bromide.

**Table 1:** Primer pairs used for PCR amplification of mRNAs from syncrip immunoprecipitations

	**Forward**	**Reverse**
Cdc42	ATGCAGACAATTAAGTGTGTTGTTGTGGA	TCATAGCAGCACACACCTGCGGCTCTTCTT
N-WASP	ATGAGCTCCGTCCAGCAG	TCAGTCTTCCCACTCATCATC
Arp2	GGAGTTGGTGTTGCTGAAT	TAGTAGACCCTCCAGAAAGC
Arp3	CAATCCTTGGAAACTGCTA	CCATTTTGACCCATCTGTA
GAPDH	GACCCCTTCATTGACCTC	CCAAAGTTGTCATGGATG
CT45	ATGACCGATAAAACAGAGAAGG	ACAAGTCTCGTCTCATACAT

## Competing interests

The authors declare no conflict of interest.

## Authors’ contributions

HJH, OJ, SAK and DK designed the study and drafted the manuscript. AK, NS, ML and HJH conducted the experiments. All authors read and approved the manuscript.

## Supplementary Material

Additional file 1: Figure S1 Subcellular localization of CT45 and down-regulation of CT45 by RNA interference in U266B1 myeloma cells. (**A**) Immunofluorescence staining of cytospin preparations of U266B1 cells with mab Ki-A10. DAPI was used for DNA staining. (**B**) Down-regulation of CT45 using CT45 siRNA was analyzed by Western blotting in whole cell lysates 24, 48, 72, 96 and 144 hours after transfection. (**C**) A BrdU incorporation assay was done to determine the effect of CT45-down-regulation on the proliferation of U266B1 cells. (**D**) Cell cycle analyses were performed by flow cytometry 72 h after transfection with scrRNA or CT45 siRNA using propidium iodide. Histograms for scrRNA- or CT45 siRNA-treated cells are shown in the upper panels. A quantification using the indicated regions is depicted in the lower panel. Down-regulation of CT45 had no apparent impact on proliferation or cell cycle progression of U266B1 myeloma cells. Click here for file
