# Supplementary material for: Down-regulation of the cancer/testis antigen 45 (CT45) is associated with altered tumor cell morphology, adhesion and migration
Source: Cell Commun Signal. 2013 Jun 10;11:41. doi: 10.1186/1478-811X-11-41 (PMC3689639; doi:10.1186/1478-811X-11-41)
Supplement: Additional file 1: Figure S1 — Subcellular localization of CT45 and down-regulation of CT45 by RNA interference in U266B1 myeloma cells. (A) Immunofluorescence staining of cytospin preparations of U266B1 cells with mab Ki-A10. DAPI was used for DNA staining. (B) Down-regulation of CT45 using CT45 siRNA was analyzed by Western blotting in whole cell lysates 24, 48, 72, 96 and 144 hours after transfection. (C) A BrdU incorporation assay was done to determine the effect of CT45-down-regulation on the proliferation of U266B1 cells. (D) Cell cycle analyses were performed by flow cytometry 72 h after transfection with scrRNA or CT45 siRNA using propidium iodide. Histograms for scrRNA- or CT45 siRNA-treated cells are shown in the upper panels. A quantification using the indicated regions is depicted in the lower panel. Down-regulation of CT45 had no apparent impact on proliferation or cell cycle progression of U266B1 myeloma cells. [file 1478-811X-11-41-S1.docx]

**Additional information**

**Down-regulation of the cancer/testis antigen 45 (CT45) is associated with altered tumor cell morphology, adhesion and migration.**

Anja Koop^1^, Nadia Sellami^2^, Sabine Adam-Klages^1^, Marcus Lettau^1^, Dieter Kabelitz^1^, Ottmar Janssen^1^, Hans-Jürgen Heidebrecht^1,2^

^1^Institute for Immunology and ^2^Institute for Pathology, University Hospital Schleswig-Holstein Campus Kiel, Arnold-Heller-Str. 3, 24105 Kiel, Germany

**Additional Figure S1:** Subcellular localization of CT45 and down-regulation of CT45 by RNA interference in U266B1 myeloma cells. **(A)** Immunofluorescence staining of cytospin preparations of U266B1 cells with mab Ki-A10. DAPI was used for DNA staining. (B) Down-regulation of CT45 using CT45 siRNA was analyzed by Western blotting in whole cell lysates 24, 48, 72, 96 and 144 hours after transfection. (C) A BrdU incorporation assay was done to determine the effect of CT45-down-regulation on the proliferation of U266B1 cells. (D) Cell cycle analyses were performed by flow cytometry 72 h after transfection with scrRNA or CT45 siRNA using propidium iodide. Histograms for scrRNA- or CT45 siRNA-treated cells are shown in the upper panels. A quantification using the indicated regions is depicted in the lower panel. Down-regulation of CT45 had no apparent impact on proliferation or cell cycle progression of U266B1 myeloma cells.
